# Analysis of the TGF**β** functional pathway in epithelial ovarian carcinoma

**DOI:** 10.1054/bjoc.2001.1950

**Published:** 2001-09

**Authors:** K M Francis-Thickpenny, D M Richardson, C C van Ee, D R Love, I M Winship, B C Baguley, G Chenevix-Trench, A N Shelling

**Affiliations:** Department of Obstetrics and Gynaecology, Research Centre in Reproductive Medicine, National Women’s Hospital, Auckland; School of Biological Sciences, University of Auckland, Auckland; Department of Molecular Medicine, University of Auckland, Auckland; Auckland Cancer Society Research Laboratories, University of Auckland, Auckland, New Zealand; The Queensland Institute of Medical Research, c/o Royal Brisbane Hospital Post Office, Herston, QLD 4029, Australia

**Keywords:** TGFβ, ovarian cancer, genetic analysis

## Abstract

Epithelial ovarian carcinoma is often diagnosed at an advanced stage of disease and is the leading cause of death from gynaecological neoplasia. The genetic changes that occur during the development of this carcinoma are poorly understood. It has been proposed that IGFIIR, TGFβ1 and TGFβRII act as a functional unit in the TGFβ growth inhibitory pathway, and that somatic loss-of-function mutations in any one of these genes could lead to disruption of the pathway and subsequent loss of cell cycle control. We have examined these 3 genes in 25 epithelial ovarian carcinomas using single-stranded conformational polymorphism analysis and DNA sequence analysis. A total of 3 somatic missense mutations were found in the TGFβRII gene, but none in IGFRII or TGFβ1. An association was found between TGFβRII mutations and histology, with 2 out of 3 clear cell carcinomas having TGFβRII mutations. This data supports other evidence from mutational analysis of the PTEN and β-catenin genes that there are distinct developmental pathways responsible for the progression of different epithelial ovarian cancer histologic subtypes. © 2001 Cancer Research Campaign http://www.bjcancer.com


*British Journal of Cancer* (2001) **85**(5), 687–691
© 2001 Cancer Research Campaign
doi: 10.1054/ bjoc.2001.1950, available online at http://www.idealibrary.com on IDEAL

http://www.bjcancer.com

### Analysis of the TGFβ functional pathway in epithelial ovarian carcinoma

**KM Francis-Thickpenny[1], DM Richardson[1,4], CC van Ee[1], DR Love[2], IM Winship[3], BC Baguley[4], G Chenevix-Trench[5] and AN Shelling[1]**

[1]Research Centre in Reproductive Medicine, Department of Obstetrics and Gynaecology, National Women's Hospital, Auckland; [2]School of Biological Sciences, University of Auckland, Auckland; [3]Department of Molecular Medicine, University of Auckland, Auckland; [4]Auckland Cancer Society Research Laboratories, University of Auckland, Auckland, New Zealand; and [5]The Queensland Institute of Medical Research, c/o Royal Brisbane Hospital Post Office, Herston, QLD 4029, Australia

**Summary** Epithelial ovarian carcinoma is often diagnosed at an advanced stage of disease and is the leading cause of death from gynaecological neoplasia. The genetic changes that occur during the development of this carcinoma are poorly understood. It has been proposed that IGFIIR, TGFβ1 and TGFβRII act as a functional unit in the TGFβ growth inhibitory pathway, and that somatic loss-of-function mutations in any one of these genes could lead to disruption of the pathway and subsequent loss of cell cycle control. We have examined these 3 genes in 25 epithelial ovarian carcinomas using single-stranded conformational polymorphism analysis and DNA sequence analysis. A total of 3 somatic missense mutations were found in the TGFβRII gene, but none in IGFRII or TGFβ1. An association was found between TGFβRII mutations and histology, with 2 out of 3 clear cell carcinomas having TGFβRII mutations. This data supports other evidence from mutational analysis of the PTEN and β-catenin genes that there are distinct developmental pathways responsible for the progression of different epithelial ovarian cancer histologic subtypes. © 2001 Cancer Research Campaign http://www.bjcancer.com

**Keywords**: TGFβ, ovarian cancer, genetic analysis

Ovarian cancer is often diagnosed at advanced stages of the disease. Despite improvements in chemotherapy and surgical treatment, the overall 5-year survival rate for patients with epithelial ovarian cancer remains at only 35% (Landis et al, 1998). Overall, the molecular changes that underlie the initiation and development of this tumour are poorly understood. A greater understanding of these complex molecular events could lead to improvements in screening techniques and may ultimately provide new targets for chemotherapeutic drugs and gene therapy.

The transforming growth factor-β (TGFβ) family is a group of structurally related polypeptides with diverse functions. There are at least 3 highly homologous TGFβ isoforms in humans: TGFβ1, TGFβ2 and TGFβ3. TGFβ1 is the best-characterized TGFβ isoform, and is known to regulate cell proliferation and differentiation, and can inhibit or stimulate cell growth in different cell types (Yan et al, 1994). TGFβ1 is also known to have an important role in extracellular matrix deposition, cell adhesion, angiogenesis, and immune function (Polyak, 1996). In most cell types, TGFβ is a potent inhibitor of cell growth, arresting cell cycle progression in late $G_1$ phase. This growth suppression is associated with the increased expression or activities of several cyclin-dependent kinase inhibitors, including p15, p21 and p27 (Rich et al, 1999). Critical mutations in the TGFβ pathway should also lead to the loss of the ability of the cell to undergo apoptosis. TGFβ is therefore a putative tumour suppressor gene and diminished responsiveness to TGFβ is a common feature of epithelial cancers.

TGFβ1 is secreted from most cells in its latent form, by the non-convalent association with latency-associated protein (LAP). LAP is a homodimer formed from the pro-peptide region of TGFβ1, and is in turn linked to the latent TGFβ-binding protein (LTBP). Latent-TGFβ1 must undergo cleavage from this complex in order to become activated. LTBP targets latent TGFβ1 to the extracellular matrix, where it interacts with the insulin-like growth factor II receptor (IGFIIR), itself a putative tumour suppressor gene (Munger et al, 1997). It is thought that IGFIIR orientates latent-TGFβ1 for proteolytic cleavage. Once activated, homodimeric TGFβ1 binds to the constitutively active TGFβ receptor type II (TGFβRII). This binding is necessary for recruitment and activation of TGFβ receptor type I (TGFβRI). The TGFβRII phosphorylates specific serine residues immediately upstream of the serine-threonine kinase of TGFβRI, resulting in its activation. An anchor protein called Smad anchor for receptor activation (SARA) then recruits Smad2 and Smad3 to the activated TGFβRI (Tsukazaki et al, 1998). Smad2 and Smad3 become transiently associated with, and phosphorylated by, the activated TGFβRI. This association results in the formation of a complex with Smad4. This Smad complex migrates to the nucleus, and recruits other transcription factors and stimulates the expression of specific target genes. The escape from its role in growth inhibition may allow TGFβ to act in tumour cells to promote tumourigenicity through its other functions, such as by the stimulation of angiogenesis, extracellular matrix deposition and immunosuppression. Therefore defects in the TGFβ pathway might be expected to be associated with cancer development, and mutations in the coding sequence of mature TGFβ1 have been reported in ovarian (Cardillo et al, 1997a), breast (Cardillo et al, 1997b), and colon

*Received 11 January 2001*
*Revised 14 May 2001*
*Accepted 15 May 2001*

*Correspondence to:* AN Shelling cancer (Cardillo and Yap, 1997).

IGFIIR has been proposed as a putative tumour suppressor gene due to its ability to bind and degrade the mitogen IGF-II, promote activation of the growth inhibitor TGFβ, and regulate the targeting of lysosomal enzymes (Jirtle et al, 1994). Loss of heterozygosity (LOH) at the IGFRII locus at 6q26-27 is common in hepatocellular carcinomas and point mutations occur in the remaining IGFIIR allele in 25% of hepatocellular carcinomas with LOH, supporting the role of IGFIIR as a tumour suppressor gene (de Souza et al, 1995a, 1995b). LOH at the IGFIIR locus is also a common occurrence in ovarian cancer (Cooke et al, 1996), and appears to be an early event in tumourigenesis development (Chenevix-Trench et al, 1997), but mutations in this gene have not been reported in epithelial ovarian carcinoma.

Both TGFβRII and TGFβRI are transmembrane serine/threonine kinase receptors, and epithelial cells lacking them are completely unresponsive to the inhibitory response of TGFβ1 (Chen and Derynck, 1994). Several studies have shown that insensitivity to the growth inhibitory effects of TGFβ are most commonly due to alterations of the TGFβRII gene. Loss of expression of TGFβRII protein is a frequent event in prostate cancer and reintroduction of wild-type TGFβRII into prostate cancer cells lacking TGFβRII restores the growth inhibitory response by exogenous TGFβ1 (Gerdes et al, 1998; Guo and Kyprianou, 1998). In their study of ovarian cancer, Lynch et al (1998) found mutations in 6 of 24 samples (25%) in TGFβRII. These mutations included 4 missense mutations within the highly conserved serine/threonine kinase domain, 1 missense mutation within the transmembrane domain, and a single frameshift mutation in the poly(A) microsatellite region. Mutations in TGFβRII have also been reported in squamous carcinoma of the head and neck (Garrigue-Antar et al, 1995; Wang et al, 1997).

A recent report by Wang et al (2000) looked at components of the TGFβ signal transduction pathway, and screened for mutations in TGFβRI, TGFβRII, Smad2 and Smad4. Unlike Lynch et al (1998), they did not find exonic mutations in TGFβRII. However, a frameshift mutation in exon 5 of TGFβRI in 10 of 32 tumour samples (31.3%) was identified. This mutation was associated with the absence of TGFβRI expression.

It has been proposed that IGFIIR, TGFβ1, and TGFβRII function as a unit, and that somatic loss of function mutations in any 1 subunit could lead to disruption of the TGFβ1 growth inhibitory pathway and subsequent loss of cell cycle control. We screened for mutations in these 3 genes in 25 epithelial carcinomas. If mutations to these genes were identified, this would suggest that the TGFβ1 inhibitory pathway is important in the development of epithelial ovarian cancer and if these mutations are mutually exclusive in individual cancers, it would support the hypothesis that these genes act as a functional unit (Haber and Fearon, 1998).

### METHODS

### Patient DNA samples

Tumour samples from 25 women with various stages of ovarian cancer were analysed. The tumours comprised a range of histologies: 3 were mucinous, 3 endometrioid, 3 clear cell, 15 serous and 1 of mixed histology. There were 4 Stage 1, 2 Stage 2, 16 Stage 3 and 3 Stage 4 invasive adenocarcinomas. All patients were staged at laparotomy, in accordance with the recommendations of the International Federation of Gynaecology and Obstetrics (FIGO). Germline (from peripheral blood) and tumour DNA was isolated as described previously (Chenevix-Trench et al, 1992).

### Polymerase chain reaction (PCR)

PCR primers for the amplification of genomic DNA were either designed using the DNASTAR Lasergene programme or, in the case of TGFβ1 exon 7b (Cardillo and Yap, 1997) and TGFβRII (Vincent et al, 1996), derived from the literature (Table 1). Exons 8, 9, 27, 28, 31, 33, 34, 40, 47 and 48 of IGFRII, exons 5, 6 and 7 of TGFβ1 and exons 3, 5 and 7 of TGFβRII were examined. Standard reaction mixtures contained 0.2 mM dNTPs, 0.2 μM primers, 1 × PCR buffer, and 2.5 units Qiagen *Taq* DNA polymerase. Primer pairs were generally optimized by systematically varying annealing temperatures and number of cycles for standard and 'touchdown' PCR protocols (Roux, 1995), as well as by varying final concentrations of $MgCl_2$ and the addition of Q solu-

**Table 1** PCR primers: sequences and sizes of PCR products

| Gene | Exon | Forward primer 5′→3′ | Reverse primer 5′→3′ | Product size |
|---|---|---|---|---|
| IGFIIR | 8 | TTTTTATTTTGCTTCTTTCAC | ACTTGGCCCCTACTGTCC | 278 |
| | 9 | GCTGTAGTTTGGGCTGGTGTTTC | CCGGAGGGTCTGATTGTGGT | 285 |
| | 27 | AAGGGGACAACTGTGAGGTGAA | TGCAACAAAAGGAAAACGACAAA | 252 |
| | 28 | GGGAGGCCAGGGATACTTTGTC | AGCAGCCTGAGGGTGGGGAAGAA | 305 |
| | 31 | AGTTCTGTTCTAGCCTGGGGAGTC | CAGGTGAATCGGATGGTGGTTGA | 235 |
| | 33 | GTCCCCATCCTCCACCCTACA | TTCCTGGCTCTGCTCATCCTTTTT | 249 |
| | 34 | TGTTTTCTCCGCCTTTCCCTTGTG | TCTCCCGTGCCCATCTGAAAACTC | 313 |
| | 40 | CTACCGGACATCCAGCATCATA | GAGCCAACCATCGTCAAGCAGTCT | 305 |
| | 47 | TTGGGTCAGTTTTGTGGGGTTTTA | ACGGGTGTTGCTTTCCTTTCTG | 303 |
| | 48 | GGGCTCACGTGGTCTCTGCTGTT | CATTGCTCTGTGCGTTTCTCA | 294 |
| TGFβ1 | 5 | TCCCCTATCCCCTGACTCCCACAC | CTCCATCCAGGCTACAAGGCTCAC | 203 |
| | 6 | GCCCCTCCCTGCCCCTGAT | ACCTTGCTGTACTGCGTGTCC | 194 |
| | 7a | GCCCGCCGCCCGCAGGTC | GAGTGGGGGAACGTCAGGGATGGA | 374 |
| | 7b | ACGAGCCTGAGCCCTGA | CAGGTCGCCCTGTACAA | 203 |
| TGFβRI | 5 | GCCCAACCGAAATGTTAATTC | GGTAGAACTGCTTATAGAAT | 221 |
| TGFβRII | 3 | TGCAATGAATCTCTTCACTC | CCCACACCCTTAAGAGAAGA | 240 |
| | 5 | GGCAGCTGGAATTAAATGATGGGC | TGCTCGAAGCAACACATG | 255 |
| | 7 | CCAACTCATGGTGTCCCTTTG | TCTTTGGCAATGCCCAGCCTG | 250 |

 *© 2001 Cancer Research Campaign*

BJOC 01-1950 687-691 20/8/01 3:16 pm Page 689

tion. Positive (β-globin) and negative (no template DNA) controls were included in each PCR run, and amplification of a single band was confirmed by agarose gel electrophoresis and ethidium bromide staining.

### Single-stranded conformation polymorphism (SSCP) analysis

The PCR products were diluted 1/10 with sterile water. Equal volumes of diluted sample and 2 × formamide loading buffer were heated to 95˚C for 3 min to denature the samples, and immediately placed on ice to prevent DNA strands from reannealing. A 2 µl aliquot of each sample was electrophoresed alongside non-denatured and denatured controls. Altogether 4 different SSCP conditions were used, with polyacrylamide gels consisting of 8% or 14% polyacylamide, with or without glycerol (10%). Setting agents were 15 µl 25% (w/v) ammonium persulphate and 15 µl TEMED for every 10 ml of non-denaturing gel. Electrophoresis was performed at room temperature (20–24˚C) using 0.5 × TBE running buffer. Mini gels were electrophoresed at 40 mA for 10 min, then typically for 50 mA for 2 h. The DNA was visualized using silver staining in standard conditions. The gels were washed, transferred to Whatman 3 mm filter paper, dried and stored.

### DNA sequencing

Tumour samples that gave abnormal SSCP mobility shifts were re-amplified by PCR from the original DNA sample and purified with the Wizard PCR Preparations DNA Purification System (Promega). Genomic DNA from blood samples were also sequenced, to confirm that any observed sequence variation was tumour specific. Sequencing was performed with the forward primer using an Applied Biosystems Model 377 automated sequencer. Any putative mutations identified by DNA sequencing were confirmed by re-sequencing a sample of the original DNA with forward and reverse primers.

### RESULTS

PCR primers for the IGFIIR gene were designed to cover the exons that have been reported to date to contain somatic mutations (exons 27, 28, 31, 40, and 48) (IGFIIR mutation database: http://www.geneimprint.com/), and for those exons for which flanking intronic sequences were available (exon 8, 9, 33, 34 and 47). PCR primers for the TGFβ1 gene were designed to cover the coding region of the mature protein product, namely exons 5, 6 and 7. In the case of exon 7, primers described in the literature were also used (Cardillo and Yap, 1997). In the case of the TGFβRII gene, exons 3, 5 and 7 were targeted for analysis because they have been found to be mutation hotspots (Vincent et al, 1996).

SSCP analysis was carried out on 25 tumour and normal samples. TGFβRII was found to contain point mutations in 3 tumours, but not in the corresponding germline DNA (Figure 1, Table 2). The mutations were all in exon 7, and all 3 were missense mutations in stage 3 tumours. No mutations were found in IGFIIR or TGFβ1. A significant association was identified between TGFβRII mutation status and histology (Fisher's exact test, $P = 0.029$). Of the 3 mutations identified, 2 of were found in 3 clear cell tumours examined, but in only 1 (a serous tumour) out of 22 tumours of other histological types that were examined.

### DISCUSSION

It has been proposed that the IGFIIR, TGFβ1, and TGFβRII genes act as a functional unit to negatively regulate cell proliferation. Somatic loss of function mutations in any one of these genes could lead to disruption of the TGFβ growth inhibitory pathway and loss of cell cycle control. As mutations in any one gene within the functional unit would be sufficient to disrupt this pathway, there would be little selective pressure to mutate the other genes and so mutations would be expected to be mutually exclusive in individual cancers (Haber and Fearon, 1998).

We have identified somatic mutations in the TGFβRII gene in 3 out of 25 (12%) tumours but no mutations were found in the IFGIIR or TGFβ1 genes. The TGFβRII amino acid sequence is highly conserved between humans, mice and chickens and all 3 missense mutations occurred at conserved residues (Table 3). The Glu$\Delta^{515}$Asp substitution results in a conservative amino acid change and occurs within the functionally important kinase domain, where point mutations have been previously identified (Lynch et al, 1998; Garrigue-Antar et al, 1995). The Arg$\Delta^{528}$Pro substitution also occurs within this kinase domain, resulting in a non-conservative amino acid change, replacing a positively charged residue with an apolar, uncharged one. X-ray crystallography of other serine/threonine kinases suggests that this highly conserved arginine and preceding conserved glutamate are essential for kinase function (Zhang et al, 1994). The Asn$\Delta^{564}$Tyr

A B

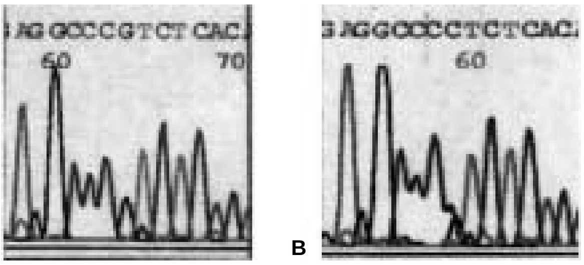

**Figure 1** DNA sequence electropherogram. Panels A and B are sequence electropherograms from normal and tumour DNA from sample 14. It shows a region of exon 7 of the TGFβRII. The sequence indicates a CGT to CCT nucleotide change

**Table 2** Somatic mutations of TGFβRII identified in ovarian carcinomas

| Gene | Exon | Sample | Nucleotide changes | Amino acid change | Domain | Comment |
|---|---|---|---|---|---|---|
| TGFβRII | 7 | 19 | GAG→GAC | Glu$\Delta^{515}$Asp | Kinase | Conservative mutation |
| | 7 | 14 | CGT→CCT | Arg$\Delta^{528}$Pro | Kinase | Non-conservative mutation |
| | 7 | 8 | AAC→TAC | Asn$\Delta^{564}$Tyr | Adjacent kinase | Non-conservative mutation |

Three missense mutations were identified in the TGFβRII gene.

*© 2001 Cancer Research Campaign* 

**Table 3** Conservation of TGFβRII protein sequences between human, mouse and chicken. The mutant sequence shows the human exon 7 TGFβRII sequence from residue 509–566, with the 3-point mutations identified in human ovarian carcinomas highlighted by shading. The amino acid positions that are not conserved in the mouse or chicken, compared to human protein sequence, are highlighted by shading

| TGFβRII type | Protein sequence |
|---|---|
| Mutant | GIQMVCDTLTECWDHDPEAPLTAQCVAERFSELEHLDRLSGRSCSEEKIPEDGSLYTT |
| Human | GIQMVCETLTECWDHDPEARLTAQCVAERFSELEHLDRLSGRSCSEEKIPEDGSLNTT |
| Mouse | GIQIVCETLTECWDHDPEARLTAQCVAERFSELEHMDRLSGRSCSQEKIPEDGSLNTT |
| Chicken | GIQMVCETLIECWDHDPEARLTAQCVAERFSEFKHHDKLSGRSCSEEKIPEDGSVTT |

substitution occurs outside the kinase domain, but also results in a non-conservative amino acid replacement, exchanging a polar uncharged residue to an aromatic non-polar one. These types of changes are well known to affect the protein folding of many kinases, often leading to changes in kinase activity or substrate recognition (Taylor et al, 1992). Functional assays would be necessary to confirm the consequences of these mutations, but given their nature and location it is likely that they contribute to tumorigenesis.

TGFβRII mutations were significantly associated with histology, and mutations occurred in 2 of the 3 clear cell carcinomas examined. This type of ovarian tumour represents a distinct histological type where the overall prognosis for patients is poor in comparison with other histologies. The outcome is largely due to the poor response to conventional platinum-based chemotherapy such as cisplatin (Goff et al, 1996; Makar et al, 1995; Tanmella et al, 1998).

This study identified tumour-specific TGFβRII sequence alterations in 3 out of 25 (12%) patient samples. Lynch et al (1998), who performed SSCP analysis of the entire coding region of the TGFβRII gene, found TGFβRII mutations in 6 out of 24 (25%) epithelial ovarian carcinomas. All 4 missense mutations reported by Lynch et al (1998) resulted in non-conservative amino acid substitutions. The relevance of these figures to ovarian carcinoma overall is unclear because both studies examined relatively small numbers of tumours, and SSCP has a sensitivity level of approximately 70% in detecting mutations (Orita et al, 1989). This is of particular concern if specific common, missense mutations are refractory to detection by SSCP. In addition, we chose to screen known gene hotspots, and it is possible that mutations are located within unscreened exons. Therefore the observed mutation frequencies should be regarded as minimum estimates. Lynch et al (1998) also performed immunohistochemistry (IHC) to examine the expression of TGFβRII protein. A total of 15 out of 25 (62%) samples demonstrated complete loss or a significant decrease of TGFβRII gene expression. This data suggests that factors other than mutations in the coding region of this gene may contribute to loss of protein expression, and thus to tumorigenesis. Such mechanisms could include promoter hypermethylation, genomic mutations outside the coding region affecting transcriptional activation or errors in post-translational modification.

We were unable to identify mutations in exon 5 of the TGFβRI gene in 11 ovarian cancer cell lines by direct sequence analysis, and 20 tumour samples by SSCP. This variant causes a significant change in DNA sequence, which should have been detected by SSCP or DNA sequence analysis. The discrepancies between our study and Wang et al (2000) remain unexplained. As larger studies emerge, correlations between specific histology and mutations in each of the genes may be seen.

Other members of the TGFβ functional pathway may also be targets of mutation since other proteins, in addition to IGFIIR, TGFβ1, TGFβRI and TGFβRII, are required. Candidates would include Smad2, Smad4 and p21, which are all mutated in various types of cancer (ten Dijke and Heldin, 1999). However, the recent study by Wang et al (2000) would suggest that mutations in Smad2 and Smad4 are rare. While mutations occur relatively frequently in various members of the pathway, there is currently little data to determine whether there is also decreased gene expression of these genes, due to epigenetic mechanisms such as promoter hypermethylation, as has been seen for other tumour suppressor genes. Examination of key components of the TGFβ functional pathway in a large series of patient samples would provide vital information regarding the importance of this pathway in the progression of ovarian cancer. Demonstration of a relationship between TGFβRII mutations and clear cell carcinomas of the ovary would suggest that the TGFβ pathway is important in the development of this distinct and aggressive subtype of epithelial ovarian carcinoma. The possibility of distinct developmental pathways for epithelial ovarian cancer subtypes has been suggested previously. Somatic mutations in the PTEN (Obata et al, 1998) and β-catenin (Wright et al, 1999) genes are found most frequently in endometrioid ovarian tumours, suggesting that these genes may promote differentiation toward this specific subtype. The results presented here provide further evidence for the role of distinct developmental pathways in the progression of epithelial ovarian cancer subtypes.

### ACKNOWLEDGEMENTS

Funding was provided by the Cancer Society of New Zealand, the University of Auckland Research Committee, and the Auckland Medical Research Foundation.

### REFERENCES

Cardillo MR, Yap E and Castagna G (1997a) Molecular genetic analysis of TGF-β1 in ovarian neoplasia. *J Exp Clin Cancer Res* **16**(1): 49–56

Cardillo MR, Yap E and Castagna G (1997b) Molecular genetic analysis of TGFβ1 in breast cancer. *J Exp Clin Cancer Res* **16**(1): 57–63

Cardillo MR and Yap E (1997) TGF-β1 in colonic neoplasia: a genetic molecular and immunohistochemical study. *J Exp Clin Cancer Res* **16**(3): 281–288

Chen R-H and Derynck R (1994) Homomeric interactions between type II transforming growth factor-beta receptors. *J Biol Chem* **269**: 22861–22874

Chenevix-Trench G, Leary J, Kerr J, Michel J, Kefford R, Hurst T, Parsons P, Friedlander M and Khoo S-K (1992) Frequent loss of heterozygosity on chromosome 18 in ovarian adenocarcinoma which does not always include the DCC locus. *Oncogene* **7**: 1059–1065

Chenevix-Trench G, Kerr J, Hurst T, Shih Y-C, Purdie D, Bergman L, Friedlander M, Sanderson B, Zournazi A, Coombs T, Leary JA, Crawford E, Shelling AN, Cooke I, Ganesan TS, Searle J, Choi C, Barrett JC, Khoo S-K and Ward B (1997) Analysis of loss of heterozygosity and *KRAS2* mutations in ovarian neoplasms: clinicopathological correlations. *Genes Chromosomes Cancer* **18**: 75–83

Cooke I, Shelling AN, Le Meuth VG, Charnock ML and Ganesan TS (1996) Allele loss on chromosome arm 6q and fine mapping of the region at 6q27 in epithelial ovarian cancer. *Genes Chromosomes Cancer* **15**: 223–233

 *© 2001 Cancer Research Campaign*

De Souza AT, Hankins GR, Washington MK, Fine RL, Orton TC and Jirtle RI (1995a) Frequent loss of heterozygosity on 6q at the mannose 6-phosphate/insulin-like growth factor II receptor locus in human hepatocellular tumors. *Oncogene* **10**: 1725–1729

De Souza AT, Hankins GR, Washington MK, Orton TC and Jirtle RL (1995b) *M6P/IGF2R* gene is mutated in human hepatocellular carcinomas with loss of heterozygosity. *Nature Genet* **11**: 447–449

Garrigue-Antar L, Muñoz-Antonia T, Antonia SJ, Gesmonde J, Vellucci VF and Reiss M (1995) Missense mutations of the transforming growth factor β type II receptor in human head and neck squamous carcinoma cells. *Cancer Res* **55**: 3982–3987

Gerdes MJ, Larsen M, McBride L, Dang TD, Lu B and Rowley DR (1998) Localisation of transforming growth factor-beta(1) and type II receptor in developing normal human prostate and carcinoma tissues. *J Histochem Cytochem* **46**(3): 379–388

Goff BA, Sainz de la Cuesta R, Muntz HG, Fleischhacker D, Ek M, Rice LW, Nikrui N, Tamini HK, Cain JM, Greer BE and Fuller AF (1996) Clear cell carcinoma of the ovary: a distinct histologic type with poor prognosis and resistance to platinum-based chemotherapy in stage III disease. *Gynecol Oncol* **60**(3): 412–417

Guo Y and Kyprianou N (1998) Overexpression of transforming growth factor (TGF) betal type II receptor restores TGF-betal sensitivity and signaling in human prostate cancer cells. *Cell Growth Differ* **9**(2): 185–193

Haber DA and Fearon ER (1998) The promise of cancer genetics. *Lancet* **351** (Suppl II): 1–8

Hollstein M, Sidransky D, Volgelstein B and Harris CC (1991) p53 mutations in human cancers. *Science* **253**: 49–53

Jirtle RL, Hankins GR, Reisenbichler H and Boyer IJ (1994) Regulation of mannose 6-phosphate/insulin-like growth factor-II receptors and transforming growth factor beta during liver tumor promotion with phenobarbital. *Carcinogenesis* **15**(8): 1473–1478

Landis S, Murray T, Bolden S and Wingo PA (1998) Cancer statistics, 1998. *CA Cancer J Clin* **48**: 6–29

Lynch MA, Nakashima R, Song HJ, Degroff VL, Wang D, Enomoto T and Weghorst CM (1998) Mutational analysis of the transforming growth factor beta receptor type II gene in human ovarian carcinoma. *Cancer Res* **58**(19): 4227–4232

Makar AP, Baekelandt M, Trope CG and Kristensen GB (1995) The prognostic significance of residual disease, FIGO substage, tumour histology, and grade in patients with FIGO stage III ovarian cancer. *Gynecol Oncol* **56**(2): 175–180

Munger JS, Harpel JG, Gleizes PE, Mazzieri R, Nunes I and Rifkin DB (1997) Latent transforming growth factor-beta: structural features and mechanisms of activation. *Kidney Int* **51**(5): 1376–1382

Obata K, Morland SJ, Watson RH, Hitchcock A, Chenevix-Trench G, Thomas EJ and Cambell IG (1998) Frequent Pten/Mmac mutations in endometrioid but not serous or mucinous epithelial ovarian tumors. *Cancer Res* **58**(10): 2095–2097

Orita M, Iwahana H, Kanazawa H, Hayashi K and Sekiya T (1989) Detection of polymorphisms of human DNA by gel electrophoresis as single-strand conformation polymorphisms. *Proc Nat Acad Sci USA* **86**: 2766–2770

Polyak K (1996) Negative regulation of cell growth by TGF β. *Biochim Biophys Acta* **1242**: 185–199

Rich JN, Zhang M, Datto MB, Bigner DD and Wang X (1999) Transforming growth factor-β-mediated p15(INK4B) induction and growth inhibition in astrocytes is SMAD3-dependent and a pathway prominently altered in human glioma lines. *J Biol Chem* **274**: 35053–35058

Roux KH (1995) Optimisation and troubleshooting in PCR. In: Diffenbach CW and Dveksler GS (eds), *PCR Primer: a Laboratory Manual*, CSHL Press: New York

Tammella J, Geisler JP, Eskew PN and Geisler HE (1998) Clear cell carcinoma of the ovary: poor prognosis compared to serous carcinoma. *Eur J Gynaecol Oncol* **19**: 438–440

Taylor S, Knighton DR, Zheng J, ten Eyck LF and Sowadski JM (1992) Structural framework for the protein kinase family. *Ann Rev Cell Biol* **8**: 429–462

ten Dijke P and Heldin C-H (1999) An anchor for activation. *Nature* **397**: 109–111

Tsukazaki T, Chiang T, Davidson A, Attisano L and Wrana J (1998) SARA, a FYVE domain protein that recruits Smad2 to the TGFβ receptor. *Cell* **95**: 779–791

Vincent F, Hagiwara K, Ke Y, Stoner GD, Demetrick DJ and Bennett WP (1996) Mutation analysis of the transforming growth factor beta type II receptor in sporadic human cancers of the pancreas, liver, and breast. *Biochem Biophys Res Commun* **223**: 561–564

Wang D, Song H, Evans JA, Lang JC, Schuller DE and Weghorst CM (1997) Mutation and downregulation of the transforming growth factor beta type II receptor gene in primary squamous cell carcinomas of the head and neck. *Carcinogenesis* **18**(11): 2285–2290

Wang D, Kanuma T, Mizunuma H, Takama F, Ibuki Y, Wake N, Mogi A, Shitara Y and Takenoshita S (2000) Analysis of specific gene mutations in the transforming growth factor-beta signal transduction pathway in human ovarian cancer. *Cancer Res* **60**: 4507–4512

Wright K, Wilson P, Morland S, Campbell I, Walsh M, Hurst T, Ward B, Cummings M and Chenevix-Trench G (1999) β-catenin mutation and expression analysis in ovarian cancer: exon 3 mutations and nuclear translocation in 16% of endometrioid tumours. *Int J Cancer* **82**: 625–629

Yan Z, Winawer S and Friedman E (1994) Two different signal transduction pathways can be activated by transforming growth factor β1 in epithelial cells. *J Biol Chem* **269**(18): 13231–13237

Zhang F, Strand A, Robbins D, Cobb MH and Goldsmith EJ (1994) Atomic structure of the MAP kinase ERK2 at 2.3å resolution. *Nature* **367**: 704–711

*© 2001 Cancer Research Campaign*
